# The Design and Analysis of the Fabrication of Micro- and Nanoscale Surface Structures and Their Performance Applications from a Bionic Perspective

**DOI:** 10.3390/ma17164014

**Published:** 2024-08-12

**Authors:** Haohua Zheng, Jiawei Liu, Yake Qiu

**Affiliations:** Architecture and Design College, Nanchang University, Nanchang 330031, China

**Keywords:** bionics, ultrafast laser, micro- and nanoscale surface structures, surface property analysis, laser surface manufacturing technology

## Abstract

This paper comprehensively discusses the fabrication of bionic-based ultrafast laser micro–nano-multiscale surface structures and their performance analysis. It explores the functionality of biological surface structures and the high adaptability achieved through optimized self-organized biomaterials with multilayered structures. This study details the applications of ultrafast laser technology in biomimetic designs, particularly in preparing high-precision, wear-resistant, hydrophobic, and antireflective micro- and nanostructures on metal surfaces. Advances in the fabrications of laser surface structures are analyzed, comparing top-down and bottom-up processing methods and femtosecond laser direct writing. This research investigates selective absorption properties of surface structures at different scales for various light wavelengths, achieving coloring or stealth effects. Applications in dirt-resistant, self-cleaning, biomimetic optical, friction-resistant, and biocompatible surfaces are presented, demonstrating potential in biomedical care, water-vapor harvesting, and droplet manipulation. This paper concludes by highlighting research frontiers, theoretical and technological challenges, and the high-precision capabilities of femtosecond laser technology in related fields.

## 1. Introduction

Different organisms have evolved many different functional surfaces adapted to their natural environments, and these surfaces have properties that are unmatched by those of modern artificially structured materials. However, the excellent physical and chemical properties of many materials in nature are often determined by the multilayered structures of their main components at different scales [1,2,3,4]. In fact, many different types of materials exist in different organisms, but their compositions differ in spatial location and distribution patterns. Natural materials endow organisms with superior self-cleaning, low-reflectivity, adhesive, actuating, sensing, and responsive properties [5,6]. This high adaptability to the environment is achieved by the optimization of self-organized biomaterials at all levels of the multilayered structure, which enables superior functionality.

Ordered, multiscale structures with different characteristics ranging from the macroscopic to the nanoscale are very common in natural materials. In this respect, the abundance of species in nature has provided us with many new ideas for different morphologies rather than different chemical compositions, leading to different properties, such as adhesion, wear-resistance, lubrication, wetting, self-cleaning, antifouling, and antimicrobial properties [7,8,9,10]. In addition, the addition of nanoparticles to different artificially designed scaffolds can be used to control cell behaviors, such as adhesion or proliferation. Applications have been made in cutting-edge fields such as wearable devices, manned deep submersibles, and photovoltaic devices [11,12,13,14].

**Figure 1:**
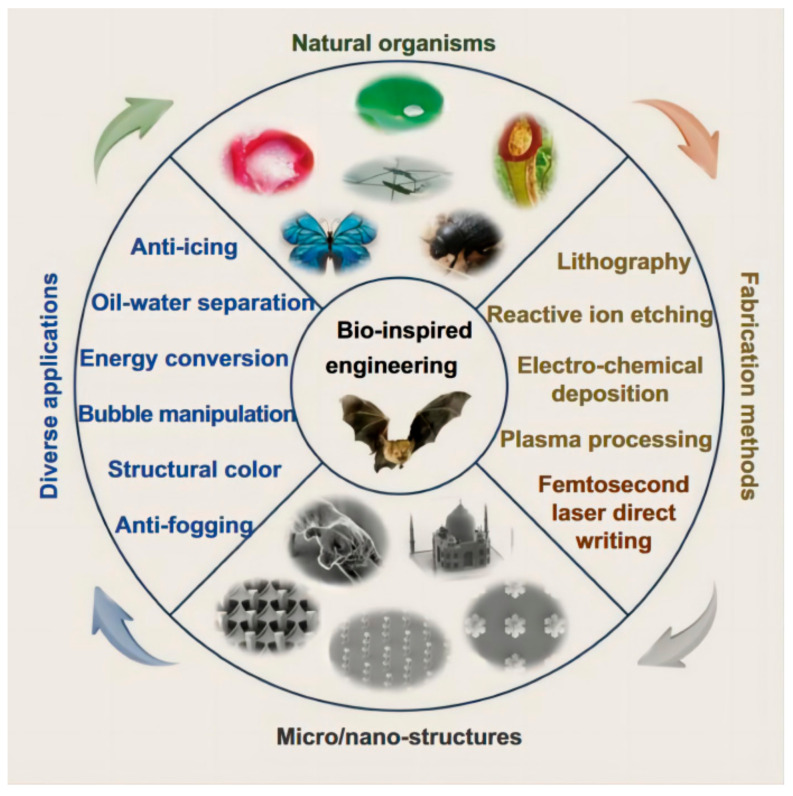
Organisms with specialized functional surfaces and their microstructures [23]. Reproduced with permission from Dong Wu. Bioinspired micro-/nanostructured surfaces prepared by femtosecond laser direct writing for multi-functional applications; published by IOP Publishing Ltd., 2020.

There are many surface structures in the natural world that can be borrowed (Figure 1), and in the development of surface science and technology, bionic research methods for designing structures with characteristics similar to those of plants and animals in the natural world are currently common. In the natural world, organisms have formed many complex surface chemical structures through evolution and have thus developed a variety of special properties necessary for survival.

Certain organisms in nature have superior surface structures, and friction-resistant drag-reducing structures are very common. The hexagonal scale configuration of pythons effectively reduces friction during crawling [15]; the V-shaped grooves on the surface of tamarisks show excellent resistance to erosion in the presence of wind and sand mixtures [16]; and the groove structure on shark skins can have an effect on friction against walls, thus reducing drag. The raised surfaces of beetles and ridges with striated elevations on the heads of ants can improve surface lubrication and reduce wear rates [17,18,19]. Organisms can evolve non-smooth surface structures such as grooves and bumps, which greatly reduce friction and drag during their movement [20,21].

The micromorphology of the surface of natural lotus leaves was characterized and analyzed using scanning electron microscopy (SEM), and it was found that a large number of irregularly arranged micropapillae were distributed on the surface of the leaves, and there were also nanoscale fluffy materials on its edge profile, which were called waxy nanowire (NW) structures, with low surface energy, and constituted a micro/nanocomposite structure [20]. Due to their surface microstructure, natural lotus leaves are self-cleaning, antireflective, and superhydrophobic [22].

Surfaces at different scales show a variety of colors due to their strong selective absorption of light at different wavelengths within the spectral range. Multiscale specially structured micro-/nanosurface materials, on the other hand, can be colored by reflecting and refracting light within specific wavelengths, capturing the light at specific wavelengths, or capturing the light altogether to achieve invisibility. Therefore, in nature, most animals rely on their antireflective surface structures to escape from their natural enemies, for example, butterfly wings, cicada wings and moth eyes, which reduce the reflectivity of incident light [23,24]. The moth’s eye is a cone configuration with hexagonal vertices, while the cicada’s wings are a periodic nanocylindrical array structure that is distinctly different from the moth’s bimodal eye. These materials are mainly based on polysaccharides (chitins) and their derivatives as basic structural units. Another example of a natural antireflective structure is the glassy-winged butterfly, whose wing surface consists of irregularly arranged micro–nano cylindrical arrays [25,26,27], as show in Table 1.

Ultrafast lasers currently have a very wide range of applications in the field of bionic design. As a new surface-processing technology, ultrafast laser technology can realize the precision design and processing of micro–nano structures, which greatly improves the accuracy of the fabrication of bionic micro–nano structures. Ultrafast laser bionanostructuring combines bionics and ultrashort pulsed laser-related technologies, which can prepare high-precision and high-quality wear-resistant, hydrophobic, and antireflective bionanostructures on metal surfaces, and provides new ideas and new processes for applications in related fields [28,29,30].

This study discusses the fabrication and performance analysis of bionic-based ultrafast laser micro–nano-multiscale surface structures. It explores the functionality of biological surface structures and achieves high adaptability through the optimization of multilayered self-organized biomaterials. By providing a detailed introduction to the application of ultrafast laser technology in bionic design, this study analyzes the advancements in the fabrication of laser surface structures. Additionally, this paper summarizes the applications of stain-resistant self-cleaning, bionic optical, wear-resistant, and biocompatible surfaces. It offers readers an in-depth understanding of the fabrication and applications of ultrafast laser micro–nano structures from a bionic perspective and proposes future research directions and potential technological improvements.

## 2. Advances in Laser Surface-Structure-Manufacturing Technology

Ultrafast lasers have been developed to a large extent in recent years, with femtosecond lasers benefiting from their extremely short pulse widths, which allow for very high peak powers to be obtained at the focal point even when the pulse energy is only microjoules or millijoules. Under strong optical field conditions, the electric field strength of the laser distorts the Coulomb potential of most neutral atoms, leading to nonlinear interactions between light and matter, such as multiphoton absorption or tunneling ionization. It has a wide range of advantages in practical applications due to its high efficiency, high precision, greenness, independence of the material and shape of the processed object, and pulse width on the order of femtoseconds (1 fs = 10–15 s), and ultrahigh peak energy (>1012 W/cm^2^). Conventional micro- and nanofabrication technologies have certain limitations, such as requiring too many machining procedures, which are difficult to operate; requiring harsh cutting conditions for cutting platforms made of special materials; and producing secondary pollution, which is unfavorable to the environment. However, how to efficiently, accurately, and simply construct novel micro- and nanostructures with multiple bioactivities is still an urgent problem to be solved [31,32,33,34].

Common ultrafast laser micromachining methods can be roughly divided into two types, namely, top-down and bottom-up methods, which include electrochemical etching, single-point ablation, line scanning, multibeam interferometry, and other [35,36,37] processing methods. Generally, this surface-structuring technique utilizes suitable laser power to directly irradiate the surface of the material, which can be processed over a large area without auxiliary means, presenting micro- and nanostructures at a certain scale [38,39]. There are also large differences in the processing mechanisms when using ultrafast lasers for different substances, which are related to the different absorption modes of photons by the substances [40]. The femtosecond laser direct writing process works by utilizing a nonlinear absorption mechanism that occurs when the femtosecond laser interacts with some materials, and this method allows for the material to be fully absorbed in the focusing region, which greatly reduces the interaction region and increases its processing accuracy [41]. The process of completing the processing through the interaction of the laser with the material and further graphicizing it can be considered laser direct writing in the general sense.

Due to its unique physical properties, ultrafast laser processing is excellent at suppressing thermal diffusion, which results in an extremely small heat-affected zone (HAZ), allowing nanoablation to be performed at subwavelengths or smaller resolutions. This enables the laser processing of structures at subwavelengths and even shorter times at the micro- and nanoscale. The high processing resolution exceeds the diffraction limit of the laser beam due to the threshold effect when using a Gaussian beam [35].

Direct laser writing (DLW) is a technology that uses a laser with a controlled scanning path to process a specific pattern on a substrate, compensating for the shortcomings of traditional contact lithography. DLW technology was first developed in the 1980s, with line widths as small as 1 µm. High-precision processing has been widely used and plays an important role. DLW enables accurate and rapid energy inputs for the fast removal of surface material from the machined part; at the same time, it significantly reduces the generation of heat-affected zones or cracks. Whether in microvias or in structures such as grooves, ultrafast lasers allow for the edges of the processed structures to be sharply flattened with a higher accuracy and resolution [42]. The typical multidimensional processing characteristics of lasers allow them to meet the requirements of both macroscale and microscale and even nanoscale manufacturing processes, allowing for the multiscale, selective, and noncontact modification of the structure and for the properties of the material to achieve manufacturing goals.

**Figure 2:**
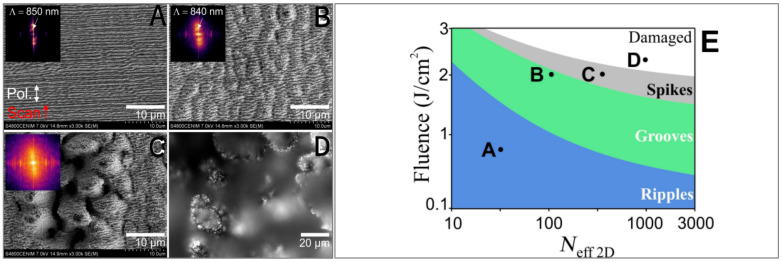
SEM images of three LIPSS structures produced under different laser irradiation conditions, distinguished fundamentally by the laser fluence and pulse number Neff_2D (parameters given in the text). (**A**) ripples with a periodicity Λy = 850 nm, (**B**) grooves, Λy = 840 nm, Λx = 2.6 μm, and (**C**) spikes. (**D**) Highly irregular (“damaged”) morphology (optical micrograph) obtained at high fluence and Neff_2D values. (**E**) Schematic distribution of the different structures found with one single scan, depending on the laser fluence and the effective number of pulses in an area (Neff_2D). The positions on this plot for the structures shown in (**A**–**D**) are represented accordingly. The scanning direction and laser polarization for all the images shown are included in (**A**) [42]. Reproduced with permission from Jan Siegel. Biomimetic surface structures in steel fabricated with femtosecond laser pulses: influence of laser rescanning on morphology and wettability; published by Beilstein-Institut, 2018.

Laser direct writing produces induced periodic surface structures (LIPSSs), which are based on the directional self-organization of materials and which allow for the fabrication of specific micro- and nanostructures in extended areas. On the surface of certain materials, a specific periodic structure is generated as a result of pulsed laser irradiation. Such periodic structures on the order of subwavelength scales can be directly shaped using laser pulses. The pulsed laser is generated by a regenerative amplification system, which expands the output laser beam and then focuses it through a column lens to obtain an elliptical focal spot, with the scanning direction being perpendicular to the laser direction, resulting in the formation of an LIPSS. For LIPSSs, the period and direction of the nanoripple structure are the main concerns for structural control (Figure 2). They are closely related to the laser’s power, polarization, etc., which is expected to realize their wide application in the fields of integrated optics and bionic micro- and nanodevices.

When multiple laser beam scans of a steel surface are performed and maintained for a certain number of pulses, they cause morphological changes that are completely different from those of the conventional single-scanning method. The number of times the laser rescanned areas that are processed during the machining process has a great influence on the degree of surface structural organization. Although this parameter greatly influences the surface morphology [43,44], there are almost no related studies. Using this method, complex bionic structures can be fabricated on the surface of 16MnCr5 gear steel (steel 1.7131). Studies carried out on different irradiation parameters to produce the desired assembled structures have shown the effect of both the laser repetition frequency and the laser injection volume on the number of times the same area is rescanned by the laser. The latter parameter is crucial for controlling the morphology and size of a given structure. As an example of structural functionality, surface wettability was chosen, and its dependence on laser-processing parameters was investigated. Contact angle measurements of water droplets placed on the surface showed that a wide range of contact angles can be obtained by choosing the appropriate irradiation parameters, such as the number of scans, and that the resulting structures from the above methods can be obtained with a great range of wettability from hydrophilic to hydrophobic to superhydrophobic [45,46].

Changes in the surface morphology of stainless steel under the action of femtosecond laser double pulses reveal the effects of parameters such as energy density, energy dose, and interpulse delay on morphological features. A short pulse interval (τ = 5 ps) favors the formation of 2D low spatial frequency laser-induced periodic surface structures (LFLs), while a longer pulse interval (τ = 20 ps) leads to 2D high spatial frequency LIPSs (HSFLs). The resulting surface patterns were analyzed and characterized for their wettability and cell adhesion properties. A relationship between surface roughness and contact angle was proposed, and it was confirmed that surface morphologies with different roughness and complexity values exhibited different wetting properties. Patterns with different spatial characteristics showed variable cell adhesion responses, suggesting that this method can be used as a strategy for preparing customized surfaces for the development of functional implants [47].

## 3. Structural Type Analysis of Micro- and Nanoscale Bionic Surfaces

### 3.1. Stain-Resistant, Self-Cleaning Surfaces

Through femtosecond laser ablation, fluorosilanization, and impregnation with inert perfluoro oil, a smooth liquid-infused surface was successfully engineered on food-grade stainless steel and tested for its antifouling properties under real industrial conditions. The resulting hydrophobic surface (with a water contact angle of 112°) exhibited extremely slippery properties (with a contact angle hysteresis of 0.6°). After a 90 min pasteurization and short water rinse test on a pilot-scale facility, no traces of milk deposition were detected, and excellent fouling-release properties were obtained for these liquid-infused surfaces [48].

Two layers of quasiperiodic self-organized structures, at the micro- and nanometer-scale, were prepared on titanium surfaces mimicking the surface of a lotus leaf using femtosecond laser ablation. The first layer consisted of large granular-raised features between 10 and 20 μm. The first layer consisted of large grains with a high degree of granularity. The second layer existed on the surface of these grains, where irregular undulations of 200 nm (or less) in width were present. The introduction of the bionic surface pattern significantly changed the surface wettability of the titanium surface. The water contact angle of the pristine surface was θ W 73 (3°), while the laser-treated titanium surface became superhydrophobic, with a water contact angle of θ W 166 (4°). The results of the study of *S. aureus* and *P. aeruginosa* interactions with these superhydrophobic substances are as follows. The interaction of *S. aureus* and *P. aeruginosa*’s interactions with these superhydrophobic surfaces at the surface–liquid interface revealed a highly selective retention pattern for two pathogenic bacteria. Although *S. aureus* cells were able to successfully colonize the superhydrophobic titanium surface without *P. aeruginosa* cells, they were able to attach to the surface (i.e., any attached bacterial cells were below the estimated lower limit of detection) [49].

Ultrafast pulsed laser micro- and nanofabrication technology was applied for the 3D biomimetic modification of material surfaces. The artificial surfaces obtained by the femtosecond laser processing of Si in a reactive gas atmosphere exhibited a roughness that mimics the graded morphology of natural surfaces at both the micrometer and nanometer scales. With the spatial control of topology, defining surface chemistry provides materials with important wetting properties that have potential applications in open microfluidics. Based on functional coatings deposited on laser-patterned 3D structures, we can realize artificial surfaces with a very low surface energy, which can lead to water repellency and self-cleaning [50].

In the study of polymer surface properties, replica poly(methyl methacrylate) (PMMA) plates were found to produce surface effects. First, periodic patterns with sufficient precision were prepared on glass plates at the microscale using a femtosecond laser. These glass plates served as master plates that could be structured over a large distance with good control of their roughness. Then, the polymer plates were obtained by propriety polymerization without any solvent and using a cast sheet process for good reproducibility and overall simplicity and speed and ease of handling, so that this environmentally friendly modification process, by introducing visual iridescent properties and greater hydrophobicity, allows us to foresee new applications for commercialized polymers [51].

Volcanic ash is a major threat to aviation safety. The softening/melting temperature of volcanic ash is much lower than the typical operating temperature of aircraft engines. Therefore, molten ash can accelerate the failure of thermal barrier coatings (TBCs). Here, inspired by natural superhydrophobic surfaces (e.g., lotus leaves), a hydrophobic thermal barrier coating made of molten volcanic ash- was developed, which offers the great possibility of eliminating the molten ash problem of thermal barrier coatings. A hierarchically structured surface consisting of microconical papillae and regularly distributed nanoparticles with an aspect ratio of 1.10 ± 0.15 was prepared on (Gd0.9Yb0.1) 2Zr2O7 (GYbZ) spheres by ultrafast laser direct writing, confirming the feasibility of bionic microstructures for the repulsion of molten volcanic ash wetting. Subsequently, bionic-structured GYb Z thermal barrier coatings were successfully prepared by plasma spraying via physical vapor deposition. The anti-molten volcanic ash properties of the designed surfaces were attributed to the nanoscale microstructure of the bionic lotus leaf, especially the simulated nanoparticle-like structure. These studies may be an important step toward the development of next-generation aero-engines resistant to the effects of harsh weather environments [52].

Highly ordered, multidirectional, and complex bionic structures were fabricated by utilizing the unique and versatile angular profile of a CV fs laser beam. Bionic surface structuring was achieved by the point-by-point and large-area scanning of radially and angularly polarized light, yielding biscale, ruffled, superhydrophobic surfaces and sharkskin-like bionic morphologies. Although the fabrication of these specialized morphologies has been demonstrated, the laser processing of CV beams has the great potential of providing a large number of complex structures and bionic surfaces. Our approach introduces new concepts for the ultrafast laser structuring of materials and can be considered an emerging laser-based fabrication technique that can be used to expand the breadth and novelty of potential applications [53].

**Figure 3:**
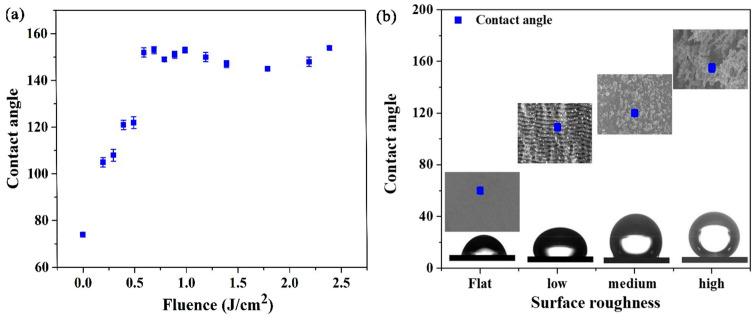
(**a**) Measured contact-angle values as a function of laser energy density. (**b**) Contact-angle values obtained from measurements of different surface roughness values at the same energy injection [55].

The wettability characteristics of LIPSSs have become a hot research topic for scholars at home and abroad. Usually, the wetting properties of liquid and solid surfaces are affected by three main factors: (1) the solid–liquid surface energy, (2) liquid viscosity, and (3) surface morphology of solids. The contact angle of the droplet has a large correlation with the surface morphology of the solid. Figure 3 shows that the surface morphology greatly affects the surface roughness and contact angle. Figure 3a shows the change in the contact angle (θM) for 15 samples measured at different laser energies. The contact angle increases with an increase in the laser energy density. Figure 3b illustrates the varying increase in the contact angle for droplets on different surface morphologies [54].

### 3.2. Bionic Optical Surfaces

The generation of self-organized nanopillar structures on fused silica (SiO_2_) through the use of ultrashort laser pulses has become a biomimetic approach to achieve omnidirectional transparent antireflective glass. The laser-induced nanostructures were selectively woven on the glass surface to mimic the spatial randomness, columnar morphology, and remarkable antireflective properties of the wings of glass-winged butterflies, Greta oto, and various cicadas. The artificial structures exhibited impressive antireflective properties in both the visible and infrared frequency ranges and were very stable over time. Accordingly, for the sp-line-polarized configuration, the reflectance of the laser-processed glass surfaces was less than 1% for different angles of incidence in the visible spectral range. However, in the near-infrared spectrum, the laser-woven glass showed greater transmittance than did the pristine glass. It is foreseen that these results will revolutionize antireflective transparent surface technology and will affect many applications ranging from glass displays to optoelectronic devices [55].

PMMA polymeric materials offer flexibility and the potential for fabricating artificial compound eye structures. The hemispherical shells of PMMA polymeric materials contain bioinspired compound eye structures consisting of approximately 5867 honeycomb hexagonal microlenses. This simple and low-cost preparation process involves femtosecond laser-enhanced wet etching and casting followed by a thermomechanical process to convert the film into a hemispherical surface. The surface was formed by optimizing the thermomechanical process parameters, and the experimental results show that the microlenses were aligned omnidirectionally on a dome with a lens diameter of approximately 85 μm and an angle of approximately 2° between the two lenses and that the individual microlenses possessed basic focusing and imaging properties. The artificial compound eye structure prepared by this method has great potential for applications [56].

Complex arrays of micro- and nanostructures of chalcogenide (ChG) glass are mass-produced by precision glass molding (PGM), which is increasingly in demand in infrared optical systems due to its excellent antireflective properties. Nickel-phosphorus (Ni-P)-coated molds are commonly used in the PGM process, and since nickel atoms tend to react with ChG glass at elevated temperatures, a novel two-step PGM process was proposed for their production. Ni-P molds are used to transfer antireflective micro- and nanostructured arrays onto silicate glass. A silicate glass with a glass transition temperature (Tg) higher than that of the ChG glass was then used as a mold to replicate the antireflective structured surface on the ChG glass. The results show that the micro- and nanostructured arrays of the ChG glass have a high molding accuracy and good infrared light transmission characteristics. Silicate glass was shown to be a suitable mold material for the ChG glass molding process. This work provides a novel PGM technique for ChG glass materials [57].

In nature, cicada wings with wide field-of-view angles and high transparency can be bioactivated to form broadband antireflective subwavelength nanostructures (ASSs), which can significantly improve the transmittance of optical devices. In this study, femtosecond laser micromachining was utilized to prepare biomimetic nanostructure arrays on optical glass. The mechanism of transmittance enhancement and the geometrical design of the artificial structures were probed at visible and near-infrared wavelengths using a rigorous coupled-wave analysis method. In addition, the height, diameter, and period of the femtosecond laser-induced ASSs were tuned by controlling the pulse energy and scanning speed of the laser. As a result, the proposed biomimetic nanostructures are expected to be widely used in glass displays due to their antireflective and hydrophobic properties in the wide-angle omnidirectional visible wavelength band [58].

### 3.3. Friction-Resistant Surfaces

A hard material surface resembling the texture of python scales significantly improves the tribological properties. Numerical models of hexagonal weave configurations with two orientations (θ = 0° and 90°) were used, and the corresponding specimens were prepared by laser grossing. The tribological properties were investigated theoretically and experimentally to examine the effects of geometric features and operating conditions. Overall, the experiments verified the trends identified in the simulations, and both showed that textured surfaces with θ = 90° can exhibit better frictional behaviors than textured surfaces with θ = 0°. In addition, compared with a smooth surface, a hexagonal textured surface with an area density of 25% reduces the friction coefficient by up to 41%. Overall, the results show that the geometrical characteristics of hexagonal weaving and operating conditions have a significant effect on the tribological performance under full-film lubrication conditions. Numerical models of hexagonal weaves with two orientations (θ = 0° and 90°) were developed using MATLAB (R2014a), and the corresponding specimens were prepared by laser burring. The tribological properties were investigated theoretically and experimentally to examine the effects of geometric features and operating conditions [13].

The genus *Tamarix* grows in arid and semiarid regions and has adapted to wind-driven conditions by evolving extremely efficient and robust erosion-resistant surface patterns. However, the details of these unique properties and their structural basis remain unexplored. In this paper, we demonstrate that tamarisk spp. surfaces suffer only minor scratching under erosion by wind–sand mixtures. The results suggest that the erosion resistance of the bionic specimens, inspired by the different surface morphologies of the tamarisk spp. surface, can be attributed to the reduced particle impact velocity by the rotating flow stream. In addition, simulations and experiments on the erosive-wear behavior of bionic centrifugal fan blades showed that V-shaped bionic surfaces exhibit good erosive wear resistance.

The unique hexagonal, bionic microstructure of alloy steel was inspired by the microstructure of the surface layer of the body of tree frogs, which provides good transport and load-bearing properties for the uniform transport of its secretions across the body surface. Furthermore, this microstructure allows for the stresses to be dispersed over the numerous hexagonal structures instead of the concentrated loads generated on their contact surfaces. In this study, a bionic microstructure combined with solid-lubricated Sn-Ag-Cu was designed to improve the tribological properties of AISI 4140 steel. The optimized parameters of the bionic texture were obtained by the RSM method. A uniform lubricant film with a thickness of 2.4 μm was formed on the surface of the microstructure, which resulted in the uniform bonding of the film to the substrate. Compared with those of the unweaved AISI 4140 steel, the optimized bionic fabrication of AISI 4140 steel—Sn–Ag–Cu—reduced the average coefficient of friction by 20.82%, the coefficient of friction fluctuation by 54.35%, and the depth of abrasion by 65.65%. The uniformly dispersed lubricant on the wear surface better isolates the direct contact of the friction partners, reduces friction and wear, and leads to a significant reduction in the phenomenon of discontinuous layers of wear marks. Therefore, the tribological properties of AISI 4140 steel can be significantly improved by optimizing the microtexture through RSM [59].

### 3.4. Biocompatible Surfaces

An organizational structure plays a crucial role in the physiological function of blood vessels. Surface-patterned films hold promise for replicating cellular arrangements, as in natural blood vessels. However, for the applications of vascular tissue engineering (TE), current surface-patterned films lack structural support for myoendothelial communication between the mesentery and endothelium. Here, we report the development of direct microperforation on surface-patterned films using a femtosecond laser for the native structural reconstruction of blood vessels. Anisotropic microcrests/grOOVes were used to surface pattern polycaprolactone (PCL) films. Direct femtosecond laser ablation further created micrometer-sized through-holes in the PCL films without invasive thermal damage to the ridges/grOOVes. After direct femtosecond laser microperforation, the PCL films exhibited improved flexibility without sacrificing the yield stress. Additionally, direct femtosecond laser microperforation resulted in PCL films with hydrophilic permeability to transport nutrient/signaling biomolecules and to allow xenogeneic cellular protrusions to enter the through-holes of physical myoendothelial contacts. Small-caliber vascular TE scaffolds based on the prepared PCL films enabled the construction of hybrid vessel walls with a multilayer arrangement of the matrix and a fused endothelium similar to that of native vascular tissues. These results suggest that direct femtosecond laser microperforation can be used as a reliable method to prepare biomimetic films with through-holes [60].

## 4. Applicational Scenarios for Bionic Surface Structures

### 4.1. Biomedicine

**Figure 4:**
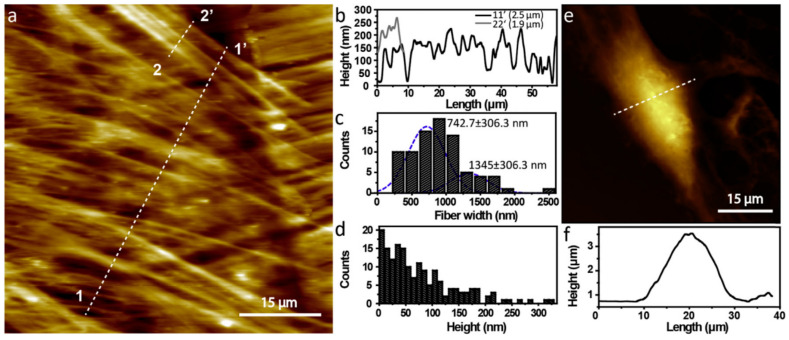
Characterization of VSMCs and HUVECs. (**a**) AFM image of the morphology of VSMCs (The dashed lines 1-1’ and 2-2’ serve as size reference scales.); (**b**) line contours of individual lines in (**a**); (**c**) diameters of the fiber structures (The blue dashed line indicates the peak diameters of the two fiber structures.); (**d**) height distribution of the cell surface; (**e**) AFM image of the morphology of HUVECs(The dashed line serves as a size reference scale.); (**f**) line contours of the white dashed lines in (**e**). [61] Reproduced with permission from Chunyong Liang, Biomimetic cardiovascular stents for in vivo re-endothelialization; published by Elsevier Ltd., 2016.

Rapid in vivo re-endothelialization using cardiovascular stents is a promising strategy for reducing cardiovascular implantation or preventing local thrombosis and restenosis. Surface-patterned endovascular prosthetic scaffolds were developed to prevent life-threatening complications (Figure 4). In this study, a femtosecond laser was used to fabricate a mimetic surface pattern of vascular smooth muscle cells (VSMCs) on a 316 L cardiovascular scaffold, which was implanted into the iliac artery of rabbits. The in vitro data demonstrate that the bionic surface pattern matched well with the morphology of VSMCs and promoted the adhesion, proliferation, and migration of the endothelial cells of human umbilical veins. In addition, the patterned surface significantly enhanced re-endothelialization. Therefore, surface bionic stents with VSMC surface patterns may be an effective method to ensure rapid re-endothelialization and potentially reduce the incidence of in-stent restenosis [61].

Controlling the interaction between materials and biological tissues is a key factor in optimizing the overall performance of implants and prostheses integrated into the body. With this aim in mind, micro- and nanoscale bionic-layered 1D and 2D surface patterns were prepared on titanium alloy surfaces using femtosecond laser processing. The experimental results show that laser irradiation promotes surface oxidation, accompanied by the formation of polarization-dependent nanoripples. Subsequently, human mesenchymal stem cells were cultured on different surface patterns to determine their response to the underlying micro- and nanostructures. A ripple morphology was shown to induce nonfouling behavior, which can be exploited in FA [62].

### 4.2. Water-Vapor Collection

Natural superhydrophilic and superhydrophobic surfaces provide inspiration for a variety of bionic designs. In particular, the combination of these two extreme wetting states with micropatterning opens up interesting applications such as water-vapor collection for Namibian desert beetles. The sheath wings of this species of beetles can be mimicked by a novel three-step fabrication method to improve the efficiency of water-vapor collection. In the first step, a two-layer hierarchical surface structure was generated on a Pyrex wafer using femtosecond laser structuring, which amplified the intrinsic wetting properties of the surface, making it superhydrophilic (with a water contact angle of <10°). In the second step, the laser-constructed surface was made superhydrophobic (with a water contact angle of >150°) by the deposition of a polytetrafluoroethylene-like polymer (CF2) n via a plasma process. In the case of the fog-collecting sheath wings of the desert beetle nanobirr, the Teflon-like coating was selectively removed by femtosecond laser ablation in the last step to simulate superhydrophilic spots on the superhydrophobic surface.

To investigate the effects on the behavior of fog collection, (super)hydrophilic, (super)hydrophobic, low-contrast and high-contrast wetting patterns were prepared on glass wafers using all reasonable combinations of these three processing steps and were exposed to fog in an artificial nebulizer device. This study revealed that the high-contrast wetting pattern had the greatest amount of fog and improved the efficiency of fog collection by nearly 60% compared to that of a pristine Pyrex glass. A comparison of the behavior of the fog collection of six samples revealed that the superior efficiency of the fog collection of surface patterns with extreme wetting contrasts is due to a combination of water attraction and water repulsion: superhydrophilic spots act as droplet aggregation zones, while the surrounding superhydrophobic region allows for rapid water transport induced by gravity. This approach enables the rapid and flexible surface functionalization of a wide range of materials, including transparent substrates, opening up possibilities for biomedical and microfluidic designs [63].

### 4.3. Droplet Manipulation

Microfluidics has an important role in biochips in particular. Despite significant advances in droplet manipulation, existing bioassays still face challenges in the microcapture of difficult-to-access samples and the immediate analysis of biological samples at low temperatures. To overcome these limitations, a droplet manipulator with self-actuation and electrical stimulation was developed using femtosecond laser micromachining and postprocessing strategies. Inspired by cactus and hogweed plants, a wedge-shaped structure with microbowl arrays and silicone oil infusion was incorporated into SES-SDM. Synergistic with ultralow (4.0 V) stimulation, these biologically inspired properties enabled SES-SDM to spontaneously and controllably deliver droplets, exhibiting the largest rapid motion (15.7 mm/s) and the longest distance (96.2 mm). Notably, the SES-SDM could operate at −5 °C without droplet freezing. Self-driven motions and electrically responsive pinning accurately captured and analyzed droplets. More importantly, the SES-SDM enabled the real-time detection of heavy metals in water through autopropulsion and electric braking. This work provides a methodology for designing a microsampling robotic arm (5–20 μL) to generate microsamples for effective bioanalysis and a strategy for microanalysis using coordinated droplet manipulation [64].

A multidroplet manipulation strategy based on the laser weaving of magnetically responsive tilted micropillar arrays allows for the easy fine-tuning of motion trajectories by varying the droplet volume and the magnetic field velocity. The unidirectional wave of the micropillar array driven by a specific magnetic field realizes the fast motion of the droplets in the horizontal direction. The bending angle of the micropillar can be quickly and reversibly adjusted from 0 to 90 degrees under the action of a magnetic field. Moreover, liquid light sources, motorized switches, and biomedical detection devices can be designed by manipulating droplets. The superiority of MSMA in the programmable manipulation of multiple droplets opens up new avenues for its application in microfluidics and biomedical engineering [65].

A flexible maskless 3D fabrication method using femtosecond lasers with phase-space shaping to prepare bionic unidirectional liquid spreading surfaces allowed for the lasers to transform from having a Gaussian distribution to having a 3D bionic structural field fraction, which further led to the preparation of toothed-ribbed erythrocyte mimic structures with needle-like ends for the unidirectional spreading of water [66].

Ultrafast pulsed laser micromachining technology can be applied to material surfaces for 3D biomimetic modifications. The artificial surface roughness obtained by the femtosecond laser processing of silicon in a reactive gas atmosphere can mimic the roughness of natural surface-graded morphology. With the spatial control of topology, defining surface chemistry provides materials with important wetting properties that have potential applications in open microfluidics. Artificial surfaces with a very low surface energy can be obtained by depositing functional membranes on 3D structures for waterproofing and self-cleaning [51].

### 4.4. Stain-Resistant Surfaces

Coating superhydrophobic coatings on the surfaces of transmission lines, insulators, and other surfaces can reduce the adhesion effects of ice and snow to achieve anti-icing effects. The larger the contact angle of the superhydrophobic coating, the smaller the rolling angle, the longer the cooling water freezing time, the lower the overall strength of icing, and the more obvious the anti-icing effect. The interception rate of supercooled water droplets and the icing adhesion strength are two important parameters for characterizing the icing properties of superhydrophobic surfaces. The interception rate of supercooled water droplets during the icing process of superhydrophobic coatings was investigated. The results show that the retention rate of supercooled water on the superhydrophobic surface is always lower than that of the RTV and uncoated glass plates, and the water retention rate shows an increasing trend of “slow growth-rapid growth-stable growth” with an increasing icing time, which suggests that the anti-icing effect of the superhydrophobic coating gradually improves with an increasing icing time. This indicates that the anti-icing effect of the superhydrophobic coating gradually decreases with an increasing freezing time [67].

Stabilized superhydrophobic and superhydrophilic surfaces are formed using laser surface micro-/nanopatterning. These are based on periodic 3D micro-/nanostructures produced over large areas on 316 L stainless steel using the fabrication of nanosecond and picosecond laser surfaces. The effects of laser processing parameters on controlling the micro-/nanomorphological characteristics of 24 different types of structures are presented. The surface roughness, surface chemistry, and wettability (via the water contact angle) of these surfaces were characterized. Aging experiments of up to 8 months were performed to analyze the durability of the prepared surfaces under three water (hot, normal, and ice-cold) conditions. Samples with superhydrophobic properties treated in the air with ps or ns lasers remained dry (water resistant) for more than 8 months when tested in ice water (0–4 °C), while those stored in hot water (80 °C) or room temperature water were allowed to dry for only 3–14 weeks. The samples treated in water had a shorter water resistance, while the samples treated with the ns laser in the air had the longest water resistance. The hybrid superhydrophobic surfaces had a stable lifetime of more than 6 months for water repellency and water diffusion [68].

Howida Kandil et al. investigated and compared the bionic remineralization of dental tissues in carious dentin by moringa-based bacteriostatic agents with femtosecond laser-activated bioactive glass particles. After receiving three different wavelengths of femtosecond laser irradiations at 390 nm, 445 nm, and 780 nm, the samples were photoactivated for 5 min using a femtosecond laser, with an average power of 300 mW, a pulse width of 100 fs, and a pulse repetition frequency of 80 Hz. The mineral content of the samples was obtained and analyzed using laser-induced breakdown spectroscopy (LIBS). The LIBS analysis was carried out using laser parameters with an average power of 215 mW, a wavelength of 532 nm, a pulse width of 10 ns, and a pulse repetition frequency of 10 Hz. The results show that most of the studied samples exhibited a relative increase in mineral content, which may enhance bionic remineralization. The use of femtosecond lasers has supported a minimally invasive approach for treating carious dentin and bionic remineralization [69].

Xiaozhe Chen et al. proposed a flexible maskless 3D fabrication method for preparing bionic unidirectional liquid-spreading surfaces using femtosecond lasers with phase-space shaping, which allowed for the laser to transform from having a Gaussian distribution to having a 3D bionic structural field fraction; as a result, further toothed-ribbed erythrocyte moss bionic structures with needle-like endings were prepared for the unidirectional spreading of water when the individual structures of *S. caninervis* were 34 (length), 8 (width), and 12 μm (height), and the flow length of a 1 μL droplet on the Si surface was 16 mm. In addition, a variety of bionic nepenthes, cactus, and moth structures were prepared on Si, SiO_2_, and Ti and demonstrated a 2D (S-shaped) bending flow on a silicon wafer over a period of 2320 ms and a measurable 3D bending flow with 120° turns on a titanium tube [68].

## 5. The Use of Bionic Surfaces for Product Designs

Compared with traditional product surfaces, flexible electric pressure sensors have a wide range of uses in artificial intelligence and new energy development trends. Based on the product design of wearable devices, a sensor can be outside a certain signal or some kind of signal can be transformed into an electrical signal that can be measured and, in accordance with a certain law, (a mathematical function law) can be transformed into a usable signal. To compensate for the shortcomings of traditional pressure sensors, lightweight, miniaturized, flexible pressure sensors that can withstand bending and that have excellent electrical properties have become one of the core popular research topics in the field of intelligent sensors in electronic skin, sports monitoring, and health monitoring. Flexible TPSs are expected to become an important part of future sensors due to their simple preparation, low cost, wide choice of materials, and high-cost performance.

**Figure 5:**
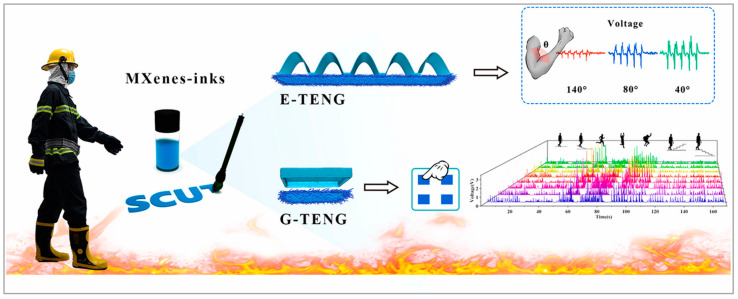
The application of the motion detection of TPSs for recognizing gestures and gaits [70]. Reproduced with permission from Saihua Jiang. Facile monitoring of human motions on a fireground by using an MiEs-TENG sensor; published by Elsevier Ltd., 2021.

The core component materials of TPSs mainly include friction materials and electrode materials. For flexible TPS materials, when choosing the materials for sensors, not only the electronegativity, sensitivity, and stability but also the wear resistance and comfort they exhibit in the application should be considered. Flexible friction electric pressure sensors based on the type of microstructure in bionic materials have wide and broad research prospects, and the various properties of the surfaces of plants and animals in nature can be applied to a variety of wearable devices. With the rapid development of the Internet-of-Things technology, it has shown great potential for applications in many fields, such as smart medicine, motion monitoring, and human–computer interactions (Figure 5).

In addition, TPSs also have better tactile-sensing abilities. Qu et al. [71] used TPSs combined with the artificial intelligence theory of machine learning to develop a smart finger that exceeds humans’ ability to sense touch. The intelligent robots could accurately recognize the type and roughness of various materials with an accuracy of 96.8%, which provides a new idea for intelligent robots to better realize the sense of touch. Li et al. [72] investigated a respiratory valve mask with a TENG-integrated friction electric pressure sensor with multiscale metal mesh electrodes, which could detect the respiratory intensity and frequency under different respiratory states in real time and could realize respiratory monitoring and recognition. With respect to respiratory monitoring and identification, Meng et al. [73] fabricated a flexible and stretchable TENG with a silver-plated polyester fabric as the electrodes, which can accurately acquire pulse wave signals from elderly and weak people and can wirelessly transmit and display patient health data via an app interface on a smartphone for the assessment and diagnosis of cardiovascular diseases.

## 6. Conclusions and Outlook

Bionic material surfaces have many biological properties, and the optimized design of surfaces to enable the realization of bionic material surfaces with excellent and long-lasting performance is a hot topic at the forefront of current research. Because various types of research studies on biomimetic surfaces are still in the early stages of development, there are many theoretical and technical problems to be solved. To realize the preparation of high-precision and high-quality bionic micro-/nanostructures on metal surfaces, the high-precision design and processing achieved by femtosecond lasers greatly simplifies the process of bionic designs and provides new theoretical and technological support for applications in related fields. The mechanism and design of wear-resistant, wetting, and antireflective bionic structure surfaces were reviewed.

Mathematical models of relevant biological surfaces and their computerized dynamic simulation schemes need to be established. Some typical stain-resistant, self-cleaning surfaces and antireflective and friction-resistant functional surfaces of aquatic plants, terrestrial plants, and animals were categorized according to their different interactions and movement modes with the environment, and the basic types were selected for mathematical analytical expressions to simulate biological surfaces through the control of the characteristic parameters of mathematical surfaces. Due to the current preparation process and limitations in the precision of equipment, femtosecond laser processing often requires multiple scanning molding steps; further optimizing the processing steps, shortening the processing time, reducing the production cost, and improving the yield are urgently needed for the development of ultrafast laser bionic surfaces. Accurate simulation data are crucial for investigating surface mechanisms, designing simulation surfaces, and accumulating low-error processing experiences. The principle of action of the laser processing of various metallic and nonmetallic materials of new types (e.g., various alloys and flexible polymers) has not yet been thoroughly investigated, and the way in which femtosecond lasers generate new structures requires more complete theoretical support. The femtosecond laser preparation of LIPSSs requires further analysis of the resulting surface geometric patterns for an exact characterization of the wettability and bio-compatibility of its material surface, facilitating the preparation of future biological cell-specific surface studies.

## Figures and Tables

**Table 1:** Biological surface-related applications of bionics, micro–nano structures, and manufacturing methods in nature.

Nature Organisms	Applications of Bionic Surfaces	Bionic Micro-/Nanostructures	Fabrication Methods
Butterfly wings, cicada wings, and moth eyes;	Structural color and energy conversion;	Biomimetic nanostructure arrays,	Lithography, reactive ion etching,
lotus leaves and rose petals;	anti-icing, oil–water separation, and anti-fogging;	periodic self-organized structures, and	electro-chemical deposition, plasma processing, and
and desert beetles and pythons	and friction-resistant surfaces	hexagonal textured surfaces	femtosecond laser direct writing

## Data Availability

Data are available upon request.
